# Exosomes loaded with ultrasmall Pt nanoparticles: a novel low-toxicity alternative to cisplatin

**DOI:** 10.1186/s12951-022-01675-4

**Published:** 2022-11-05

**Authors:** María Sancho-Albero, Ana Martín-Pardillos, Lluís Lujan, Víctor Sebastian, Jesús Santamaria, Pilar Martín-Duque

**Affiliations:** 1 Instituto de Nanociencia y Materiales de Aragón (INMA), CSIC-Universidad de Zaragoza, Zaragoza, Spain; 2 Department of Chemical Engineering and Environmental Technologies, University of Zaragoza, Zaragoza, Spain; 3 Networking Research Center on Bioengineering Biomaterials and Nanomedicine (CIBER-BBN), Madrid, Spain; 4 IIS Aragón, Zaragoza, Spain; 5 Department of Animal Pathology, University of Zaragoza, Zaragoza, Spain; 6 Instituto Universitario de Investigación Mixto Agroalimentario de Aragón (IA2), University of Zaragoza, Zaragoza, Spain; 7 Laboratorio de Microscopías Avanzadas, Universidad de Zaragoza, 50018 Zaragoza, Spain; 8 Instituto Aragonés de Ciencias de la Salud, Zaragoza, Spain; 9 Fundación Araid, Zaragoza, Spain; 10 Present Address: Department of Molecular Biochemistry and Pharmacology, Instituto di Richerche Farmachologiche Mario Negri IRCCS, 20156 Milan, Italy; 11 Present Address: Department of Surgery, University of Zaragoza Medical School, University of Zaragoza, Zaragoza, Spain

**Keywords:** Platinum nanoparticles, Exosomes, Cancer, Extracellular vesicles, Selectivity, Systemic toxicity

## Abstract

**Background:**

Platinum nanoparticles have been demonstrated to have excellent anticancer properties. However, because of the lack of specificity they must be delivered to the tumor in amounts sufficient to reach the desired therapeutic objectives. Interestingly, exosomes are considered as excellent natural selective delivery nanotools, but until know their targeting properties have not being combined with the anticancer properties of platinum nanoparticles.

**Results:**

In this work we combine the targeting capabilities of exosomes and the antitumoral properties of ultrasmall (< 2 nm) platinum nanoparticles as a novel, low toxicity alternative to the use of cisplatin. A mild methodology based on the room temperature CO-assisted in situ reduction of Pt^2+^ precursor was employed to preserve the integrity of exosomes, while generating ultrasmall therapeutic PtNPs directly inside the vesicles. The resulting PtNPs-loaded exosomes constitute a novel hybrid bioartificial system that was readily internalized by the target cells inducing antiproliferative response, as shown by flow cytometry and microscopy experiments in vitro. In vivo Pt-Exos showed antitumoral properties similar to that of cisplatin but with a strongly reduced or in some cases no toxic effect, highlighting the advantages of this approach and its potential for translation to the clinic.

**Conclusions:**

In this study, a nanoscale vector based on ultrasmall PtNPs and exosomes has been created exhibiting antitumoral properties comparable or higher to those of the FDA approved cisplatin. The preferential uptake of PtNPs mediated by exosomal transfer between certain cell types has been exploited to create a selective antitumoral novel bioartificial system. We have demonstrated their anticancer properties both in vitro and in vivo comparing the results obtained with the administration of equivalent amounts of cisplatin, and showing a spectacular reduction of toxicity.

**Supplementary Information:**

The online version contains supplementary material available at 10.1186/s12951-022-01675-4.

## Background

Cancer is a multifaceted global health issue that continues to demand new solutions and represents one of the greatest challenges in the biomedical field [1]. During the last decades, new therapies have focused on the selective and effective killing of cancer while trying to avoid damage to healthy tissues. In spite of these efforts, efficient targeting of tumor cells remains an unsolved challenge. Towards this goal, a host of smart vectors have been designed, usually decorated with targeting moieties (antibodies, peptides etc.) [2, 3] and often endowed with other capabilities, such as externally or site-activated release [4, 5]. The progress in knowledge has been formidable, but delivery efficiencies remain very low, even for sophisticated multifunctional nanoparticles [6]. In the search of alternatives, a new generation of so-called Trojan Horse delivery vectors is being developed, including tumor homing cells and extracellular vesicles (e.g. exosomes) as main tools. Indeed, exosomes (soft extracellular vesicles produced by the cells, 50 to 150 nm in size) have significant biological content, including DNA, RNA and proteins, and thanks to the specific composition of their membranes, are able to target desired organs[7–10] giving them great potential as selective delivery vectors. Theragnostic nanomaterials have been also incorporated in these vesicles especially in applications against cancer [8, 9, 11–27]. In this case, exosomes provide nanomaterials with a natural and robust delivery vehicle with high blood circulation time, low immunogenicity and ability to target specific cells or tissues [28, 29].

Since 1965, when cisplatin was discovered, organometallic molecules based on platinum have attracted attention due to their electronic, catalytic and therapeutic properties [30–33]. Cisplatin, together with other Pt-based molecules such as carboplatin, nedaplatin and oxaliplatin [34–36] are considered a cost-effective standard therapy for many types of cancer including lung, breast, ovarian, head and neck, cervical and sarcomas [37]. Platinum complexes are antiproliferative compounds whose mechanism involves covalent interactions with the nitrogen bases of the deoxyribonucleic acid (DNA), leading to secondary structures capable of stopping the transcription and replication of tumoral cells, causing their apoptosis [38]. However, a variety of adverse reactions have been described, related to neuro-, nephritic, hematic and immune toxicity and their use is therefore restricted by their severe and dose-limiting side effects [39]. As a consequence, recurrence and resistance to the treatments frequently occur, preventing complete remission. To overcome the strong toxicity problems related to cisplatin and its analogues, liposomes, polymeric and co-polymeric nanoparticles (NPs) have been used as delivery vectors, achieving some reduction in the toxicity compared with the naked drugs thanks to the increase of accumulation in cancer tissues [40, 41]. Also, Pt nanoparticles (PtNPs) have been explored, in an attempt to replicate the antitumoral effects of cisplatin [42]. PtNPs have been proposed for a variety of biomedical applications in cancer and beyond, on account of their excellent catalytic, optical and chemical properties [43]. It has been described that PtNPs can induce apoptosis mediated cell death, inhibiting the replication of cancer cells [44]. Bendale et al., reported the dose-dependent cytotoxic effect of PtNPs, causing an inhibition of the 66% of tumor growth in SCID mice after 13 days of their administration [45]. Porcel et al., demonstrated the synergistic antitumoral effects of combining PtNPs with radiotherapy [46]. Gehnrke et al., observed the in vitro cytotoxic effect of PtNPs in human colon cancer cells (HT29 and Caco-2) and attributed it to DNA damage and the alteration of the redox intracellular balance [47]. However, in spite of their promising properties, PtNPs (like any other therapeutic agent) still need to reach the site of action, meaning in this case that they must be delivered to the tumor in amounts sufficient to reach the desired therapeutic objectives.

Here we show that it is possible to combine the anticancer properties of PtNPs and the improved selectivity afforded by exosomes. To this end, we have optimized an in situ reduction procedure to obtain for the first time ultrasmall PtNPs inside exosomes. Using carbon monoxide (CO) as a mild reductant, PtNPs with a size under 2 nm were formed from a metallic precursor of Pt (H_2_PtCl_6_). The procedure leaves intact the key properties of exosomes and allows their use as a delivery vector. The antitumoral effect of this novel hybrid system has been demonstrated both in vitro and in vivo, comparing the results obtained with the administration of equivalent amounts of cisplatin, and showing a spectacular reduction of toxicity.

## Methods

### Cell culture

Human placental mesenchymal stem cells (hpMSCs) were purchased to Cellular engineering Technologies (CET (Coraville, IA, USA)). Metastatic murine skin melanoma cells (B16-F10 cells) and glioblastoma cells (U251-MG) were provided by cell services from Cancer Research-UK. They were grown in Dulbecco’s modified Eagle’s medium (DMEM, Biowest, France) supplemented with 10% fetal bovine serum (FBS, GIBCO, USA), 1% penicillin/streptomycin and 1% amphotericin (Biowest, France). For culturing hpMSCs, 5 µg mL^−1^ of FGF-2 growth factor (PeproTech, USA) were also added to the cell culture media. hpMSCs were maintained under hypoxic conditions whereas U251-MG cells and B16-F10 cells were cultured under and normoxic conditions.

To obtain the culture media free of exosomes, they were depleted from FBS by ultracentrifugation (100,000*g*, 8 h, 4 °C).

### Exosome purification

For exosome purification, cell lines were seeded in cell culture plates until they reached the 80% of confluence. After that, the cell culture medium was replaced by medium free of exosomes and cells were maintained for another 48 h. Then, Exos^B16−F10^, Exos^hpMSCs^ and Exos^U251−MG^ were isolated by serial centrifugation cycles from cell culture supernatants of source cells. In order to remove cell remaining debris, supernatants were centrifuged at 2000*g* during 20 min at 4 °C. For the separation of the microvesicles, samples were then centrifuged during 1 h at 10,000*g* at 4 °C. To finally isolate the exosomes, samples were ultracentrifuged twice for 2 h at 100,000*g* at 4 °C. The final pellets were dispensed in PBS for the following analysis.

### Synthesis of Pt-Exo

The purified exosome fraction resuspended in PBS was treated with a solution of H_2_PtCl_6_ (Mw 409.81 g/mol) at room temperature for 12 h to maximize the diffusion and incorporation of the Pt^4+^ ions in the exosomal lumen. To modulate the amount of PtNPs generated inside the exosomes, several precursor concentrations were employed (0.06; 0.12; 0.3 and 0.6 mM of H_2_PtCl_6_). The mixtures were then ultracentrifuged in order to discard the non-internalized Pt species (100,000*g*, 2 h, 4 °C). Pt-Exos^B16−F10^, Pt-Exos^hpMSCs^ and Pt-Exos^U251−MG^ were finally resuspended in PBS and treated in a 6 bar CO atmosphere during 40 min using a high-pressure Teflon-lined autoclave under gently stirring at 30 °C [48]. After the treatment, and before opening the autoclave, CO was desorbed with air to keep the resulting Pt-Exos in a neat environment.

### Characterization of Exos and Pt-Exos

Both biological and physicochemical techniques were used to characterized loaded and empty exosomes. A Pierce BCA protein assay (Thermo Fisher Scientific, USA) was performed in order to quantify the protein content in the exosomal sample according to manufacturer instructions. Western Blot was carried out in order to analyze the exosomal protein content. To do that, 10 µg of exosomes were lysed and precipitated using RIPA buffer and cold acetone (1:1 v/v) at − 20 °C for 2 h. Then, 5 µL of Laemmly buffer were added to the sample and boiled during 5 min at 95 °C. Proteins were subsequently separated using a 12% SDS-PAGE at 100 V for 2 h at room temperature and transferred to a nitrocellulose membrane at 4 °C for 4 h (300 mA). Blots were then blocked overnight at 4 ºC with 5% milk-TBS buffer. Then, they were incubated with CD9 (1:2000, Abcam) and CD91 (1:500, Santa Cruz Biotechnology) antibodies for 1.5 h. The membranes were finally washed three times with TBS Tween and incubated with the secondary antibody (anti-HRP, Sigma Aldrich) before being imaged by chemiluminescence. NTA analysis (Nanosight NS200, Malvern Panalytical) was used to determine the diameter and the concentration (expressed in particles/mL) of Exos and Pt-Exos. TEM (T20-FEI Technai transmission electron microscopy) imaging was also employed for evaluate the morphology, shape and size of the loaded and empty exosomes. The microscope was operated at 200 kV with a LaB6 electron source fitted with a SuperTwin objective lens allowing a point-to-point resolution of 2.4 A. A solution of 3% phosphotungstic acid was employed as contrast agent to visualize the exosomes. An Analytical Titan (FEI company) high resolution transmission electron microscope with a spherical aberration corrector was used for HAADF-STEM imaging at 300 kV. An elemental analysis of the Pt-Exos was carried out by EDX. Zeta potential of Pt-Exos^hpMSCs^ and Pt-Exos^U251−MG^ was measured by dynamic light scattering (DLS) at pH = 7 in a Brookhaven 90 Plus equipment using the ZetaPals software. The amount of Pt inside the vesicles was measured using microwave plasma atomic emission spectrometry (4100 MP-AES, Agilent Technologies, USA) and normalized by the total protein amount of Pt-Exos. To do that, samples were digested with 10% aqua regia (HNO_3_ + 3HCl) in 1.5 mL of dH_2_O during 2 h at room temperature. Calibrations were carried out employing a Pt standard in 10% aqua regia ranging from 0 to 10 ppm.

### Study of Pt-Exos in vitro cytotoxicity

The therapeutic properties of Pt-Exos as antiproliferative and cytotoxic agents in different cell cultures were first determined by using the Blue Cell Viability assay (Promega, United States) as previously reported. The cytotoxicity produced by 4; 2; 1; 0.5; 0.25 and 0.125 µg of Pt-Exos^hpMSCs^/100 µL was evaluated when incubating them following three different conditions. Approach 1: cells were incubated only with the Pt-Exos^hpMSCs^ (0.156 µg/µg of exosomal protein). Approach 2: cells were treated with Pt-Exos^hpMSCs^ (0.156 µg/µg of exosomal protein) containing NH_4_Cl, and approach 3: cells were treated with the Pt-Exos^hpMSCs^ (0.156 µg/µg of exosomal protein) with NH_3_ within them.

In a different experiment, 4; 2; 1; 0.5; 0.25; 0.125 µg of exosomes/100 µL loaded with different amount of PtNPs (0.560 and 0.86 µg of Pt/µg of exosomal protein) were incubated with the cells during 48 h. Exosomes loaded with 0.560 and 0.86 µg of Pt/µg of exosomal protein are named as 5x-Pt-Exos^hpMSCs^ and 10x-Pt-Exos^hpMSCs^, respectively. The results are expressed as cell viability percentage, considering as 100% the non-treated cells. Cell cultures incubated with the highest dose of exosomes were also observed by inverted conventional microscopy (Olympus IX81) in order to evaluate their morphology. All the experiments were performed in triplicate.

### In vitro cell death evaluation

To study in more detail the efficiency of Pt-Exos as an antiproliferative and a cytotoxic tool, hpMSCs were seeded onto a 96-well plate (5000 cells/well). After 24 h in culture, 4 and 2 µg of exosomes/100 µL were added (both from 5x-Pt-Exos^hpMSCs^ and 10x-Pt-Exos^hpMSCs^). Then, after 48 h, cell culture media was discarded and cells were washed twice with PBS. The cytotoxicity produced by the Pt-Exos loaded with different amounts of PtNPs was determined with the LIVE/DEAD kit (Thermo Fisher Scientific, USA) using an inverted fluorescence microscope (Olympus IX81) and following manufacturer instructions.

The selectivity and the efficiency of Pt-Exos as therapeutic vectors were quantified by flow cytometry (FACSAria BD cytometer, BD Bioscience). To determine the effect of the Pt-Exos on the proliferation rate of the different cell types, the distribution of the cell-cycle phases after the incubation of the exosomes was assessed. First, cells were seeded onto 6-well plates at a density of 250,000 cells/well. After 24 h, 2 µg of 10x-Pt-Exos^hpMSCs^ and 10x-Pt-Exos^U251−MG^ were incubated with both hpMSCs and U251-MG cells during 48 h. Then, cells were trypsinized and washed twice with PBS (1500*g*, 5 min). The final cell pellet was suspended in 200 µL of PBS and was fixed with 70% ice-cold ethanol and maintained at 4 °C in these solution for 24 h more before being analyzed under the flow cytometer. DNA staining was carried out by adding RNase A and propidium iodide to the cell solution. Control samples (non-treated cells and cells treated with the exosomes but in the absence of PtNPs within them) were also run to know the standard distribution of cells cycles in the cell lines assayed.

The analysis of cell apoptosis and necrosis was developed through a double-staining with Annexin V-FITC (5 µL) and propidium iodide (5 µL) during 15 min prior to their analysis by flow cytometry (FACSARIA BD equipment and FACSDIVA BD software).

### In vivo experiments

The procedures performed in this study were previously approved by the Project License PI07/21 and PI43/21 by the Ethic Committee for Animal Experiments from the University of Zaragoza (*Comisión Ética Asesora para la Experimentación Animal de la Universidad de Zaragoza*). Mice were fed ad libitum and their maintenance and care under specific pathogen-free conditions were performed accordingly with the Spanish Policy for Animal Protection RD53/2013 and the European Union Directive 2010/63 regarding the protection of animals destined to experimental and other scientific purposes.

In this study, six-to eight-week old female BALB/c nu/nu mice (Envigo) were used. All the animals were maintained seven days under quarantine as soon as they arrived to the animal facilities and before starting the experiments. For the induction of the xenograft tumor, animals received a subcutaneous injection of 7 × 10^6^ U251-MG cells in 200 µL of saline. To evaluate the potential weight loss or pain symptoms, animals were weighed and monitored daily. Tumor sizes were measured with a caliper every two days. The manipulation of the animals was always performed under sterile conditions in a hood.

After 11 days of tumor implantation, 100 µg of Pt-Exos were both, IV (in the tail vein) and IT, administered. Alternatively, cisplatin (a clinical approved drug) was administered as positive control to compare the effectiveness of the Pt-Exos based therapy. The animal groups included in the study are shown in Additional file 1: Table S1.

In order to compare the therapeutic effect produced by cisplatin and the Pt-Exos, the administered dose was calculated in order to inject the same amount of Pt:

$$0.86\,\text{ug}\frac{Pt}{ug\,exosome}\cdot 100\,\text{ug}\,exosomes\,administered=86\,\text{ug}\,of\,Pt\,administered$$

Taking into account the Pt present in the cisplatin molecule:

$$86\,\text{ug}\,of Pt \cdot \frac{300.01\,\text{ug}\,molecule}{78\,\text{ug}\,Pt}=330\,\text{ug} \, of \, cisplatin\, administered$$

**Fig. 1:**
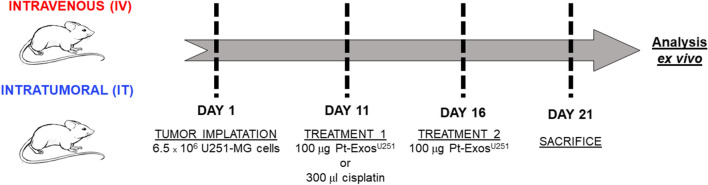
Experimental procedure followed in the in vivo experiments. Tumor implantation, both administration routes of the two therapeutic complexes (Pt-Exos^U251−MG^ and cisplatin), sacrifice of the animals and ex vivo analysis of the necropsies

In the therapeutic study, once the control animals archive the maximum tumor size approved in the Ethical Committee, the experiment was finished and the animals were sacrificed. Euthanasia of animals was performed by CO2 inhalation. Selected organs were collected from each animal for histopathological analysis and to determine the amount of Pt delivered as a consequence of treatment. For histopathological analysis the samples were fixed in 4% paraformaldehyde (Alfa Aesar) for 24 h, followed by cold 70% ethanol. The experimental plan is included in Fig. 1.

Tissue samples were then embedded in paraffin and three-micrometer sections were stained with H-E. Immunohistochemical analysis was carried out by employing human Ki-67 antibody (Agilent) with the automated immunostaining platform Autostainer Link (Dako). Sections (4–5 um) were dewaxed and rehydrated with dH_2_O and antigen retrieval was performed by heat at pH = 7 during 20 min. Tumor samples were incubated with primary Ki-67 antihuman antibody for 20 min followed by the visualization system conjugated with horseradish peroxidase (Flex/HRP system Agilent). Hematoxylin staining was used as contrast. Finally, sections were dehydrated and permanently mounted for their visualization under a conventional inverted microscope (Olympus IX81). To obtain the percentage of positive cells, Ki-67 expression was assessed in all mice groups by counting positive and negative cells in three acquisition images from all animals from each group using ImageJ software.

For the biodistribution analysis, after 11 days of tumor implantation cisplatin or Pt-Exos^U251−MG^ were injected in the tail vein and animals were sacrificed after 24 and 96 h injection. Both groups received a total amount of 86 µg Pt. In the control group tumors were induced however did not receive any Pt treatment. Pancreas, spleen, kidneys, liver, lungs, brain and tumor were collected, weighted and digested in a solution of aqua Regia during 7 days at room temperature. To evaluate platinum accumulation; the solutions were diluted (1/100) in miliQ H_2_O and total amount of the metal within the tissues was determined by ICP-MS (Perkin Elmer Elan DRC-e) in the Chemical Analysis Service from the University of Zaragoza.

### Statistical analysis

All the results are expressed as mean ± SD. Statistical analysis of the data and the significant differences among the means were analyzed by two-way analysis of variance (ANOVA) for multiple comparisons by Dunnett’s multiple comparisons test (GraphPad Software). Statistically significant differences were expressed as follows: *p < 0.05; **p < 0.01; ***p < 0.0001 and ****p < 0.00001.

## Results

### Synthesis and characterization of Pt-Exos

**Fig. 2:**
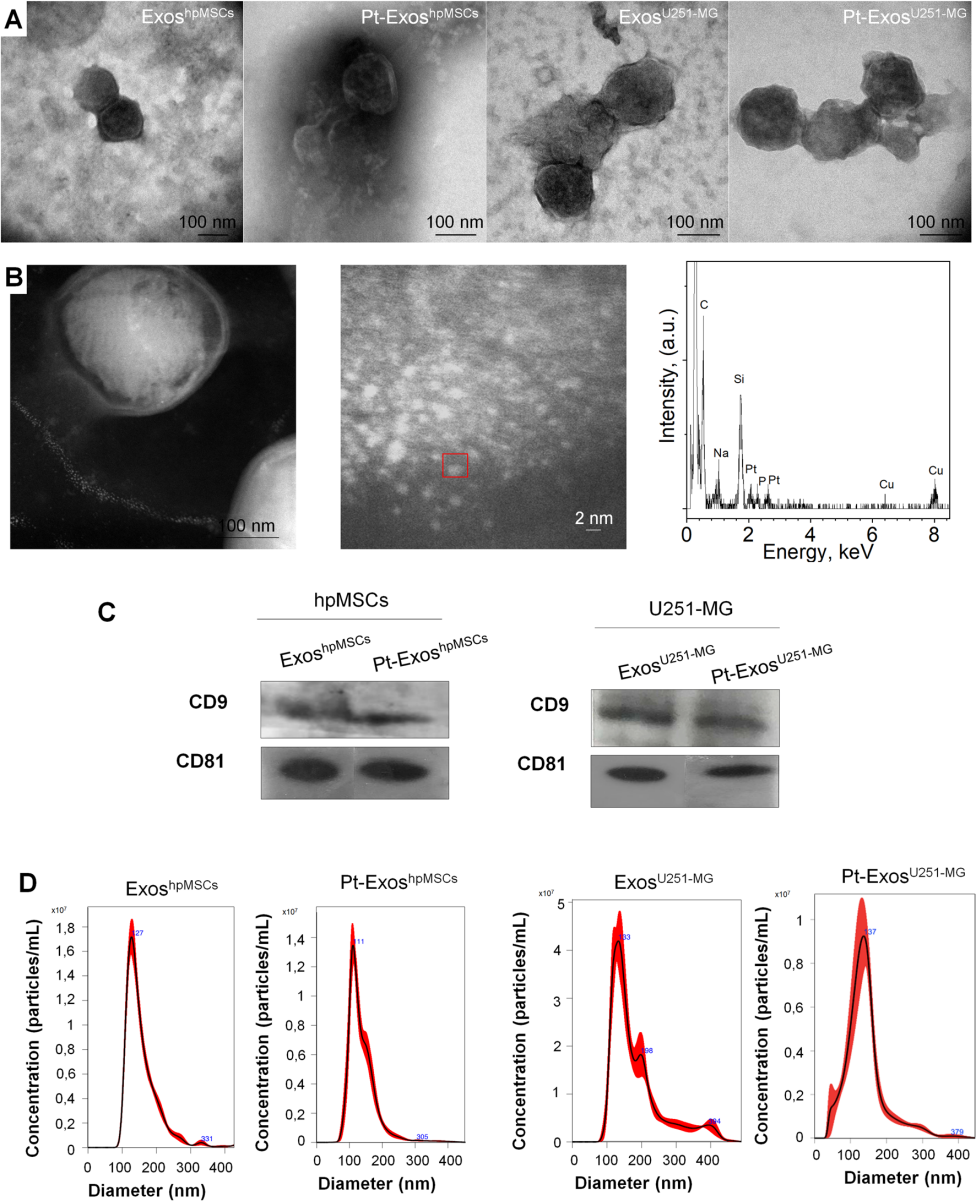
Characterization of Pt-Exos^hpMSCs^ and Pt-Exos^U251−MG^. **A** Representative TEM image of Exos^hpMSCs^, Pt-Exos^hpMSCs^, Exos^U251−MG^ and Pt-Exos^U251−MG^. Exosomes were negatively stained with phosphotungstic acid to ease imaging by a transmission electron microscopy **B** HAADF-STEM images of Pt-Exos^B16−F10^ and EDS spectrum of the red line marked area. **C** Western blots of exosome-specific biomarkers CD9 and CD81 of exosomes from hpMSC and U251-MG cells both in the presence and in the absence of PtNPs. **D** Particle size analysis of non-loaded and Pt-loaded Exos^hpMSCs^ and Exos^U251−MG^ obtained by NTA

In this work we have adapted a protocol originally developed to produce free Pt clusters using CO gas as a reductant and soft conditions (Tª > 35 °C) [49]. When the procedure is applied to create Pt nanoparticles in exosomes CO gas is solubilized at high pressure (6 bar) to promote the reduction of Pt^4+^ ions previously infiltrated by diffusion inside the exosomes. Once reduction has been achieved, CO is simply evacuated, without leaving any chemical debris in the exosomal membrane. This represents an enormous advantage compared to liquid phase chemical reductants, and is key to preserve their unique cell-specific tropism [9]. The procedure can in principle be applied to exosomes from any cell line. Figure 2A includes TEM images of exosomes from both hpMSCs (human placental mesenchymal stem cell, Exos^hpMSCs^) and glioblastoma cell lines (Exos^U251−MG^) in the absence (control) and in the presence of reduced Pt species (Pt-Exos). High-angle annular dark-field scanning transmission electron microscopy (HAADF-STEM) and energy dispersive X-ray spectroscopy (EDX) were employed to visualize the PtNPs of 1–2 nm inside exosomes (Fig. 2B). After Pt loading, the Z-potential of the exosomes was not altered. Pt-Exos^hpMSCs^ and Pt-Exos^U251−MG^ exhibited a negative surface charge (− 20.64 ± 2.90 mV and − 25.99 ± 2.62 mV, respectively) characteristic of extracellular vesicles and attributed to the negatively charged phosphate groups of their lipid membrane. To determine whether membrane proteins were altered during the chemical procedure, the presence of exosome-specific antigens CD9 and CD81 was evaluated by western blot in exosomes derived from both cell lines. As shown in Fig. 2C, similar protein expression was observed before and after Pt loading and reduction with CO, indicating that the mild reductive conditions used do not degrade membrane proteins. Finally, nanoparticle tracking analysis (NTA) revealed that both the diameter and the concentration of exosomes was not affected by the treatment with CO in the presence of the Pt precursor and the subsequent generation of the PtNPs (Fig. 2D). In the case of hpMSCs, control and Pt-loaded exosomes exhibited hydrodynamic diameters of 161.3 ± 61.9 nm and 138.9 ± 46.6 nm, respectively. For U251-MG cells, the diameters were 185.2 ± 78.5 nm and 142.0 ± 59.3 nm for Exos^U251−MG^ and Pt-Exos^U251−MG^.

The concentration of Pt inside the vesicles could be easily tuned by adjusting the concentration of the Pt precursor in the incubation media. MP-AES was used to obtain the metal loading and the results were normalized by the total protein amount of Pt-Exo, providing an average Pt concentration of 0.156; 0.306; 0.560 and 0.860 µg of Pt/µg of exosomes when they were incubated with 0.06; 0.12; 0.3 and 0.6 mM of H_2_PtCl_6_, respectively. To show the generality of the procedure, the protocol was also applied to exosomes from another cell line (B16-F10 cells). Additional file 1: Fig. S1A to S1F show the characteristics of the exosomes before and after loading with Pt nanoparticles (1.14 ± 0.34 nm, Additional file 1: Fig. S1E).

### Pt-Exos^hpMSCs^ cytotoxic and antiproliferative activity

**Fig. 3:**
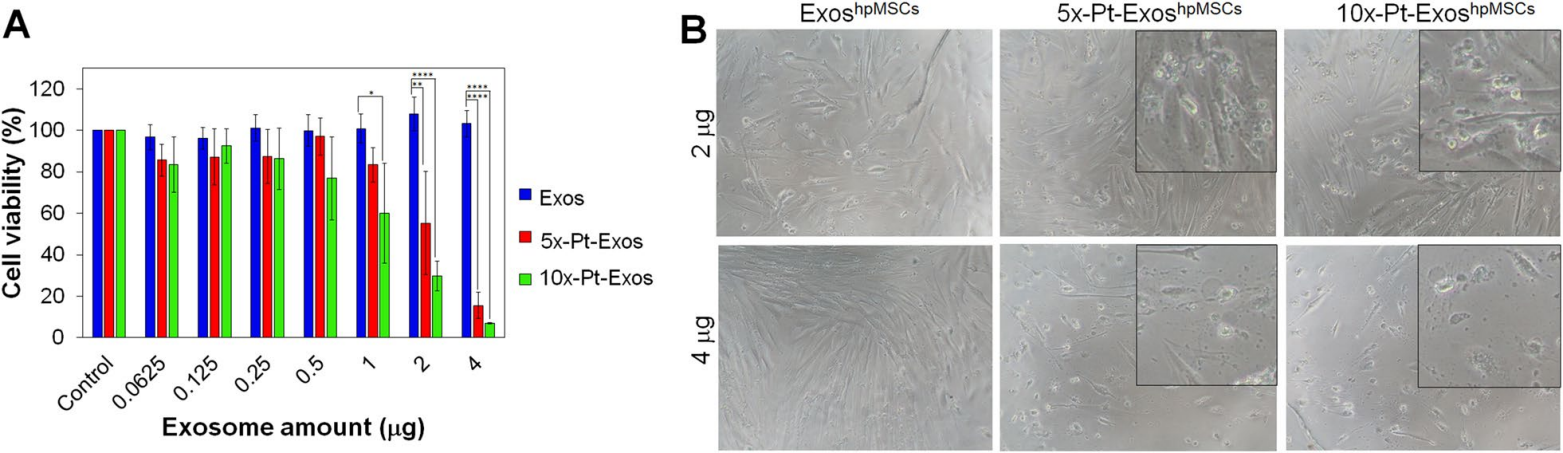
Cytotoxicity of Pt-Exos^hpMSCs^. **A** Blue Cell Viability assay results of Pt-Exos^hpMSCs^ on their parental cells when they were incubated with 5x-PtExos^hpMSCs^ and 10x-PtExos^hpMSCs^. **B** Conventional microscopy images of hpMSCs incubated during 48 h with 4 and 2 µg of both 5x-PtExos^hpMSCs^ and 10x- PtExos^hpMSCs^

To determine the efficacy of the Pt-loaded exosomes as antiproliferative agents, different concentrations of Pt-Exos^hpMSCs^ obtained at a precursor concentration of 0.06 mM were incubated with their parental cells (hpMSCs) during 48 h. Exosomes with no Pt NPs (Exos^hpMSCs^) were used as control. It can be seen that only a small and statistically non-significant decrease of cell viability was observed for the highest doses (4 and 2 µg of Pt-Exos^hpMSCs^). Then the cell viability against Pt-loaded exosomes obtained with 5 and 10 times higher precursor concentration (i.e. 0.3 and 0.6 mM of H_2_PtCl_6_), 5x-PtExos^hpMSCs^ (0.560 µg of Pt/µg of exosomes) and 10x-PtExos^hpMSCs^ (0.860 µg of Pt/µg of exosomes) was tested (Fig. 3A). Cell viabilities were smaller than 10%, 30% and 60% when incubating with 4, 2 and 1 µg of 10x-PtExos^hpMSCs^, respectively. In the case of 5x-PtExos^hpMSCs^ the cell viability percentages were 17 and 57% when 4 and 2 µg of Pt-Exos were incubated with the parental cells, respectively. The antiproliferative effects of Pt-Exos^hpMSCs^ was accompanied by a changed in cell morphology (Fig. 3B). After treatment, cells exhibited irregular shapes with a large number of vesicles and apoptotic bodies inside them, clearly more evident when cells were treated with the maximum dose (4 µg of both 5x-PtExos^hpMSCs^ and 10x- PtExos^hpMSCs^).

Fluorescence microscopy coupled to live/dead assay (Additional file 1: Fig. S2) was also used to assess the effect of increasing concentrations of Pt-loaded exosomes. Cells incubated with empty exosomes, exhibited a high viability and labelling with calcein-AM, indicated the stability of cell membranes. No red death cells marked with the EthD-1 were found. On the contrary, cell cultures exposed to 5x-PtExos^hpMSCs^ and 10x-PtExos^hpMSCs^ exhibited a dose dependent significative decrease of calcein-AM positive cells (green), with enhanced effects at the higher doses of Pt-containing exosomes and especially with exosomes loaded with higher amount of PtNPs 5x-PtExos^hpMSCs^ and 10x- PtExos^hpMSCs^.

### Selective antitumoral effect of Pt-Exos

**Fig. 4:**
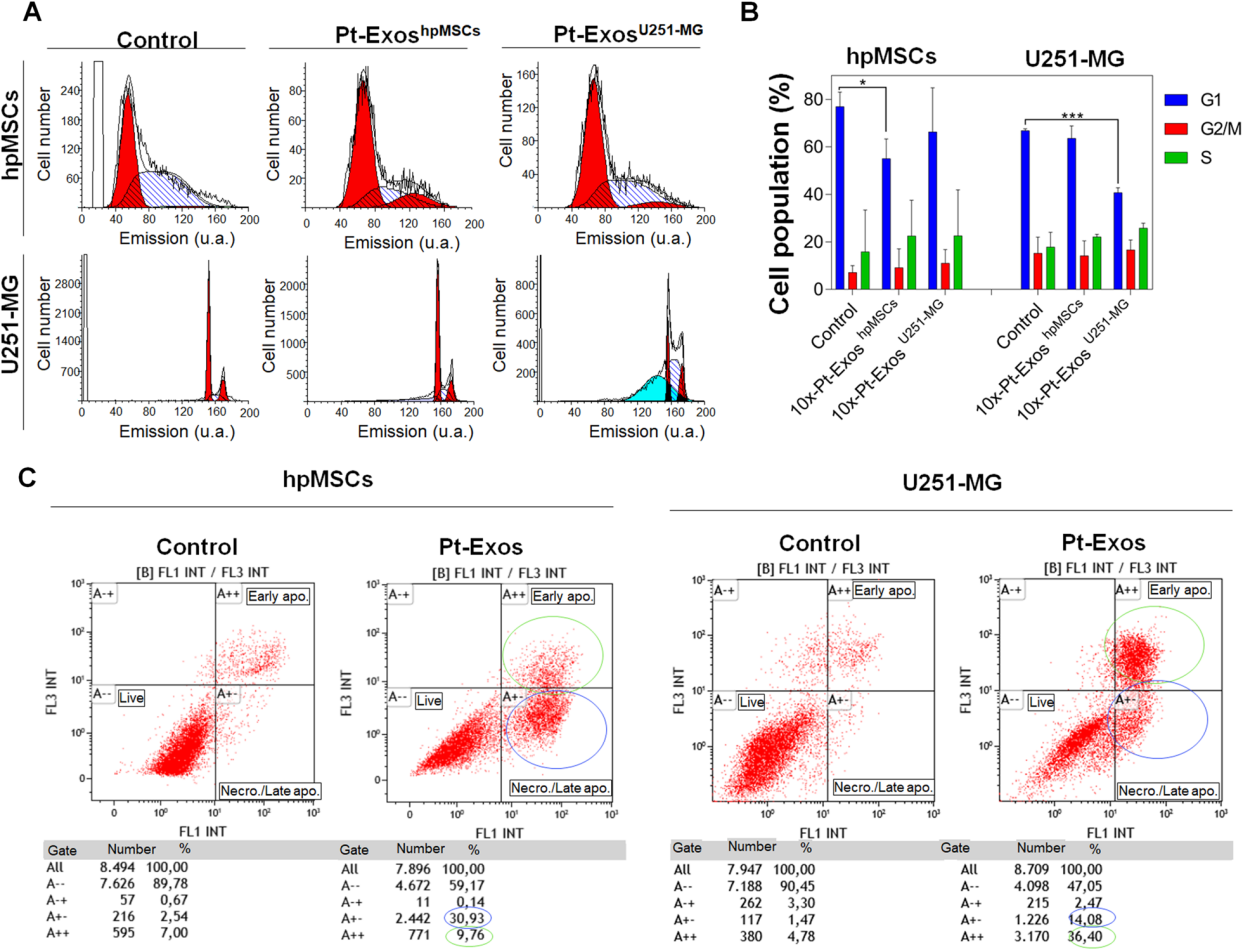
Results of flow cytometry experiments. **A** Cell cycle analysis of both hpMSCs and U251-MG cells untreated or treated with exosomes isolated from both cell lines. **B** Quantification of cell cycle phase distribution. **C** Apoptosis and necrosis results

To evaluate the selectivity of exosome mediated delivery, hpMSCs and U251-MG cells were incubated with 2 µg of 10x-Pt-Exos^hpMSCs^ and 10x-Pt-Exos^U251−MG^ during 48 h and then analyzed by flow cytometry. Cell cycle data are depicted in Fig. 4. As shown in Fig. 4A and B a significant decrease on the percentage of the cells in the G1 phase of the cycle compared with the control (untreated cells) was clearly observed when treated with Pt-loaded exosomes derived from the same parental cell line. On the contrary, no statistically significant differences were obtained in the percentage of cells in phase G1, G2/M and S when cells were incubated with exosomes produced by the other cell line. In particular, for hpMSCs, when they were incubated with 10x-PtExos^hpMSCs^, 55% of the cells were in phase G1 compared to 76.96% for non-treated cells (negative control) the cells were in phase G1. hpMSCs exposed to the presence of 10x-PtExos^U251−MG^ exhibited a 66.33% of cells in G1 phase compared to control sample. Similarly, when U251-MG glioblastoma cells were put in contact with 10x-PtExos^U251−MG^ the percentage of cells in G1 phase decreased to 40.78%, while similar percentages of cells in phase G1 were obtained for non-treated U251-MG cells and U251-MG cells treated with 10x-PtExos^hpMSCs^ (66.88% and 63.66%, respectively). This decrease of the percentage of cells in G1 phase when both cells lines were incubated with their own exosomes loaded with PtNPs led to the inhibition of cell growth and subsequently to cell death. On the contrary, when cell cultures were incubated with Pt-Exos derived from a different cell line, exosome internalization was strongly reduced and no significant differences were obtained in the percentage of cells in the cell cycle phases compared with the non-treated cells. These results confirm the fingerprint enabled by the exosomal membrane and the targeting properties of exosomes, as they are mostly internalized by their parental cells, enabling the effect of the PtNPs cargo. Figure 4C shows data on cell apoptosis/necrosis obtained by flow cytometry following incubation with Pt-Exos. It can be seen that untreated hpMSCs (negative control) exhibited a percentage of live, apoptotic and necrotic cells of 89.78%, 7% and 2.54%, respectively. When these cells were put in contact with 10x-PtExos^hpMSCs^, the population of live cells was reduced to 59% and the apoptotic and necrotic cells increased to 9.76% and 30.93, respectively. Similarly, with U251-MG cultures, 90.45% of cells were alive while 1.47% were dying by necrosis and 4.78% by apoptosis. Then, when glioblastoma cells were incubated with 10x-PtExos^U251−MG^ the cell viability decreased to 47.05% whereas the percentages of cells in apoptotic and necrotic phases increased to 36.60% and 14.08%, respectively.

### In vivo experiments

Encouraged by the high selectivity shown by the in vitro experiments of PtNPs delivery mediated by exosomes, we decided to evaluate their biodistribution and their in vivo antitumoral activity and compare it with the FDA approved drug cisplatin.

To this end, 100 µg of Pt-Exos^hpMSCs^ were administered in the tail vein of the animals. 11 days after tumor implantation, Pt-Exos^U251−MG^ or cisplatin were administered (intratumorally (IT) or intravenously (IV)). Mice were divided as indicated in the experimental section (Additional file 1: Table S1): group 1 contained non-treated (control) mice; animals of group 2 were treated with Pt-Exos^U251−MG^ IV; mice of group 3 were treated also with exosomes but IT. Group 4 and group 5 were treated with cisplatin IV and IT, respectively.

**Fig. 5:**
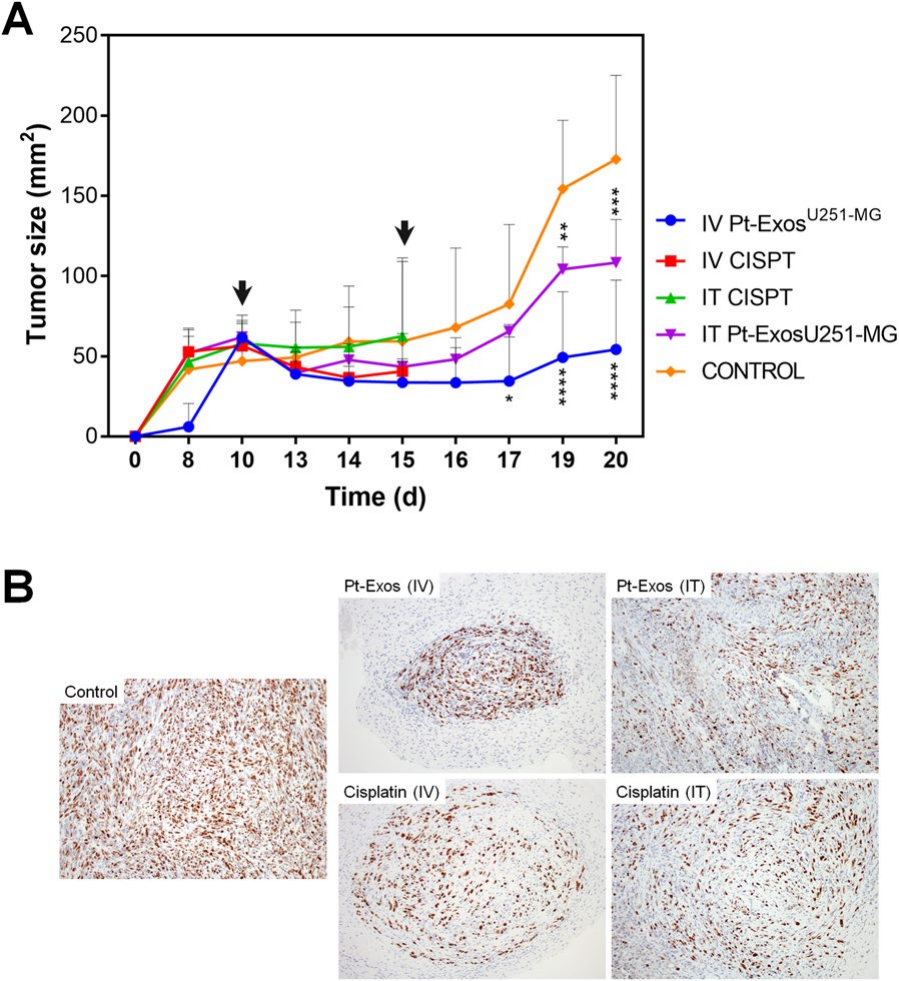
In vivo results. **A** Tumor size evolution for the control groups and the four treated groups measured with a caliper. Arrows indicate the time points for the IV or IT injections. **B** Ki-67 immunohistochemical staining of tumors from the different groups. A decrease in Ki-67 positive cells is observed in both treated groups compared with the non-treated one (control group)

Tumor development was followed until tumors in the control group reached the ethically allowed size. Figure 5A shows tumor growth for all mice groups during the first 11 days. In animals from the control group tumor size continued to grow over time. In contrast, the tumor of the animals treated both with the Pt-Exos^U251−MG^ and with cisplatin following an IV administration stopped growing and started to decrease after the first inoculation of both therapeutic materials. However, mice treated with cisplatin experimented a very significant weight decrease (higher than 20%) four days after its administration (Additional file 1: Fig. S3). Also, their general clinical and behavioral signs were strongly affected as mice were mostly inactive in a hunched position. Therefore, according to the Ethical committee approved rules, they were sacrificed at this time point. In contrast, mice of groups 3 and 4 (both treated with exosomes IV or IT, respectively) tolerated well the treatment and could receive the second dose of the treatment four days after the first administration. Again, Fig. 5A indicates that tumor growth was inhibited, demonstrating that exosomes loaded with the PtNPs not only deliver in an efficient way the PtNPs but also that the NPs preserve their antitumoral properties after their encapsulation in the vesicles. Interestingly, tumor growth inhibition was particularly effective in the case of mice treated by an intravenous approach, possibly indicating that the distribution of Pt throughout the tumor is more homogeneous when an IV injection was used, rather than IT.

The efficacy of the antitumoral treatment with exosomes loaded with the PtNPs was further studied by means of histopathological analysis. Pt-exosomes and cisplatin treated groups were compared with control (untreated) mice by hematoxylin and eosin (H-E) and Ki-67 staining. The Ki-67 protein is associated with cell proliferation, and because of this its expression it is widely employed as a biomarker for the growth rate and the aggressiveness of a tumor [50]. Histopathologic analysis of tumors from control group revealed a massive growth of tumor cells (adopting a solid pattern and exhibiting histopathologic features characteristic of cancer cells such as cellular anaplasia, nuclear enlargement, high mitotic index, basophilic nucleus, eosinophilic cytoplasm and atypical mitosis, Fig. 5B). On the contrary, in the groups treated with both Pt-Exos and cisplatin following the two different administration routes, tumor cells became more amorphous and sometimes the nucleus was difficult to distinguish. In the case of non-treated mice, an active proliferation status corresponding to mitotic cells positive for Ki-67 was clearly evidenced (Fig. 5B). On the contrary, the number of Ki-67 positive cells in Pt-Exos and cisplatin treated tumors was reduced indicating the efficacy of the treatment.

**Fig. 6:**
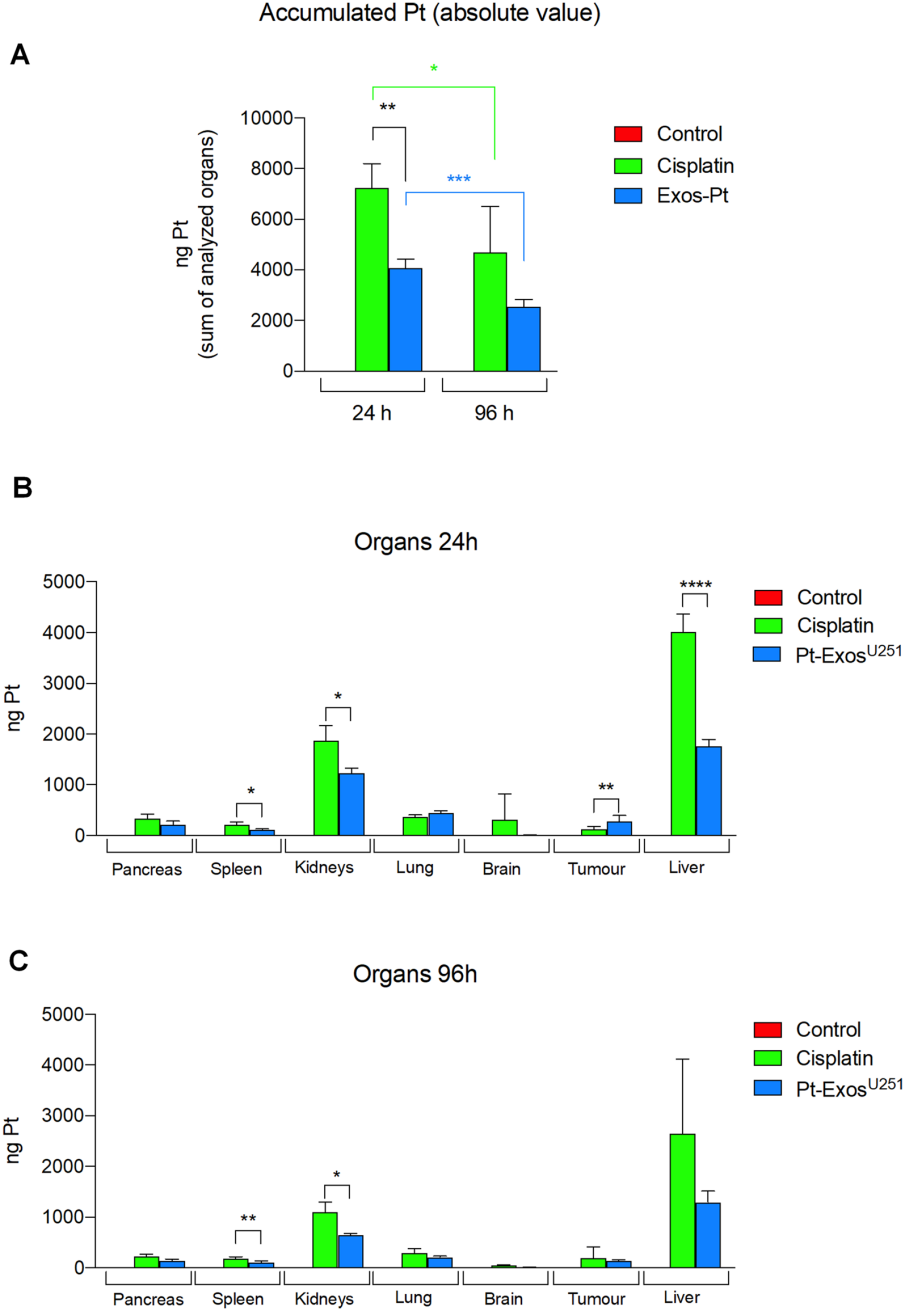
Biodistribution of Pt after IV injection of cisplatin and Pt-Exos^U251−MG^. (**A** Total amount of Pt detected per animal (sum of Pt detected in every organ). **B** Biodistribution of Pt after 24 h in analyzed organs: pancreas, spleen, kidneys, liver, lung, brain and tumor as absolute amount. **C** Biodistribution of Pt after 96 h in analyzed organs: pancreas, spleen, kidneys, liver, lung, brain and tumor as absolute amount

The biodistribution studies were carried out with mice exhibiting xenograft tumours induced using U251-MG cell line. After 11 days of tumor implantation, 100 µg of Pt-Exos^U251−MG^ loaded with 86 µg Pt or cisplatin (dose adjusted to have an equal total mass of Pt) were administered IV. The presence of Pt was evaluated in the different organs 24 and 96 h after treatment (Fig. 6). Pt was not detected in control animals, as expected. A significantly higher total amount of Pt was detected 24 h after treatment in cisplatin-treated animals. The percentage of Pt detected (or accumulated) vs Pt injected in cisplatin and Pt-Exos^U251−MG^ treated animals were 8.83% and 4.96%, respectively (Additional file 1: Fig. S4A). 96 h after injection the total amount of Pt was reduced in both treatments, cisplatin and Pt-Exos^U251−MG^ (Fig. 6A). The comparison of the Pt biodistribution data with both treatments produced very interesting findings. Thus, significantly higher amounts of Pt were detected in spleen, kidneys and liver of cisplatin-treated animals at 24 and 96 h-treatment, compared to animals treated with Pt-Exos^U251−MG^ (Fig. 6B, C). In contrast, significantly higher amounts of Pt were detected in the tumours of Pt-Exos^U251−MG^ 24 h after treatment, even showing significantly less total accumulation in Pt-Exos^U251−MG^ treated animals, after 24 h injection, the accumulation of Pt in tumours was significantly higher in Pt-Exos^U251−MG^ treated animals compared to cisplatin treated animals, 0.35% vs 0.16% respectively (Additional file 1: Fig. S4A), showing a higher effectiveness of exosome-based delivery. However, at 96 h the levels found in the tumor were comparable for both procedures (Fig. 6B).

Every analyzed organ was weighted and the relative weight was calculated in relation with the total animal weight. Animals treated with cisplatin showed a significant reduction of spleen and liver relative weight, in comparison with control animals. This alteration could be a toxic effect of the presence of Pt in these organs. The weight of spleen and liver of the Pt-Exos^U251−MG^ animals was not affected, indicating a lower toxicity (Additional file 1: Fig. S5A, B).

As already mentioned, the weight reduction was not limited to these organs. After 96 h animals treated with cisplatin reduced their total weight more than 20%, were frequently immobile, bent over themselves, and had to be sacrificed. When performing the necropsies of IV and IT cisplatin treated animals, their stomachs were considerably enlarged compared with control and with Pt-Exos treated animals (Additional file 1: Fig. S5C). However, this stomach swelling could not be associated with any microscopic lesion in the H-E stainings. The glandular part and non-glandular parts of stomachs from all animals exhibited normal microscopic features (Additional file 1: Fig. S6). These necropsy findings suggest that cisplatin treatment did not have a direct gastric impact, instead, the effect occurs on the enteric nervous system with consequent loss of gut motility. This produced a retention of food that led to stomach enlargement. It is known that peripheral nervous failure (dysautonomia) can be produced by toxic molecules, in this case, by cisplatin [51].

## Discussion

After the isolation of exosomes, the mild methodology based on CO as a reducing gas preserves the features of exosomes and does not leave any chemical debris after its use. The characterization of fresh and treated exosomes by a wide battery of both physicochemical and biological techniques did not show any significant effects of the Pt deposition treatment on exosome morphology, size or expression of exosomal proteins. Interestingly, the load of Pt could be modified by changing the concentration of the precursor, without significantly increasing the PtNPs size.

The viability of cancer and stem cells upon their exposure to Pt-loaded exosomes has been determined by incubating increasing quantities of Pt-Exos^U251−MG^ and Pt-Exos^hpMSCs^ with both cell lines during 3 days. The proliferation analysis performed by Alamar Blue, flow cytometry and LIVE/DEAD staining revealed that Pt-Exos induce a statistically significant decrease on cell viability in a dose-dependent way. Next, a cell-based assay was performed to determine whether the devices have preferential tropism for their progenitor cells by comparing their antiproliferative activity towards the different cell types. Flow cytometry experiments showed that U251-MG and hpMSCs exhibited a decreased on G1 phase when they were treated with Pt-loaded exosomes isolated from their own source cells, while the effect was clearly reduced when they were exposed to Pt-Exos with an equivalent Pt load, but where the exosomes originated from other cells. Previously reports have already shown that some antitumoral agents lead to a cell death mediated by an increase in sub-G1 phase and the subsequent decrease on G1 phase. Murad et al., have reported that the treatment of MDA-MB-231 cells with algal sulfated polysaccharide extract (ASPE) produced a decrease of the percentage of cells in phase G1 and an increase of apoptotic events. This arrest on the cell cycle was also related with the overexpression of Bac and an inhibition of Bcl-2, resulting in the activation and the unlocking of the Caspase-3 pathway and the induction of reactive oxygen species (ROS), finally leading to cell death [52]. Bendale et al., also demonstrated the growth inhibition of ovarian, pulmonary and pancreatic cancer cells upon their treatment of PtNPs in dose-dependent way and by a reduction of cells in G1 phase [45]. Regarding the mechanism of the PtNPs anticancer action, it has been shown that double stranded DNA breaks down, in a process mediated by Pt ions derived from the particles (similar to the effect caused by cisplatin) [53]. When PtNPs conjugate to the DNA, they directly interact with the double DNA helix inducing breaks in their chains. In addition, it has been reported that DNA damage is mediated by the water molecules from the solvation layer of the NPs, which are capable of generating radicals that can also damage the double helix (indirect effect). Our results demonstrate that Pt-loaded exosomes have the capacity to enter preferentially the exosome parental cells and also that the Pt-NPs generated maintain their antitumoral activity, likely related to the above discussed mechanisms. These are deployed in a selective manner thanks to the targeting properties provided by exosomes.

In vitro results prompted us to use Pt-Exos^U251−MG^ as NPs delivery tools in vivo. Our results show that the accumulation of the Pt-NPs in the tumors when their delivery was mediated by exosomes was high enough to produce a strong decrease of tumor size, comparable to that observed following the administration of the anticancer drug cisplatin at the same Pt doses. However, the toxicity of the cisplatin treatment was much higher and following the established protocols cisplatin-treated animals had to be sacrificed after 15 days of treatment. In contrast, the treatment with PtNPs loaded exosomes loaded was at least as efficient as the cisplatin treatment, but without important toxicity effects at the time intervals and doses used. Remarkably, cisplatin treated mice developed side effects in a dose and time dependent manner, and displayed cumulative toxic effects that correspond to those reported for the peripheral and central nervous systems (e.g. peripheral neuropathy, chemo brain) [54].

## Conclusions

In summary, we have developed a novel bioartificial nanoscale vector that combines ultrasmall (< 2 nm) PtNPs with exosomes created by a mild procedure that maintains key properties of the exosomes. The resulting vector brings together the targeting capabilities of exosomes and the antitumoral properties of PtNPs. We demonstrated that Pt-Exos were readily internalized by cells but that this internalization took place preferentially in the cell lines from where the exosomes were originated. In vivo experiments evidenced the efficacy of the Pt-Exos when they were IV and IT administered (their antitumoral properties were comparable or higher to those of the FDA approved cisplatin). However, the strong toxicity effects observed when cisplatin was administered were absent in the Pt-Exos treated animals. This proof of concept study opens up exciting possibilities for cancer treatments of reduced toxicity based on the exosome-mediated delivery of antitumoral agents.

## Supplementary Information

**Additional file 1**. Additional figures and Table.

## Data Availability

All data are available in the main text or the supplementary materials.
