# Supplementary material for: Exosomes loaded with ultrasmall Pt nanoparticles: a novel low-toxicity alternative to cisplatin
Source: J Nanobiotechnology. 2022 Nov 5;20:473. doi: 10.1186/s12951-022-01675-4 (PMC9636640; doi:10.1186/s12951-022-01675-4)
Supplement: Supplementary file 1 — Additional file 1. Additional figures and Table. [file 12951_2022_1675_MOESM1_ESM.pdf]

Supplementary materials

Exosomes loaded with ultrasmall Pt nanoparticles: A novel low-toxicity alternative to cisplatin

María Sancho-Albero[1,2,3,4,†], Ana Martín-Pardillos[1,2,4], Lluís Lujan[5,6], Víctor Sebastian[1,2,3,4,7,*], Jesús Santamaria[1,2,3,4,*] and Pilar Martín-Duque[3,4,8,9, ‡]

[1] Instituto de Nanociencia y Materiales de Aragón (INMA), CSIC-Universidad de Zaragoza, Zaragoza, Spain.

[2] Department of Chemical Engineering and Environmental Technologies, University of Zaragoza, Zaragoza, Spain.

[3] Networking Research Center on Bioengineering Biomaterials and Nanomedicine (CIBER-BBN), Madrid, Spain.

[4] IIS Aragón, Zaragoza, Spain.

[5] Department of Animal Pathology, University of Zaragoza, Zaragoza, Spain

[6] Instituto Universitario de Investigación Mixto Agroalimentario de Aragón (IA2), University of Zaragoza, Zaragoza, Spain.

[7] Laboratorio de Microscopías Avanzadas, Universidad de Zaragoza, 50018 Zaragoza, Spain.

[8] Instituto Aragonés de Ciencias de la Salud, Zaragoza, Spain.

[9] Fundación Araid, Zaragoza, Spain.

[†] Present Address: Department of Molecular Biochemistry and Pharmacology, Instituto di Richerche Farmachologiche Mario Negri IRCCS, 20156, Milano, Italy.

[‡] Present Address: Department of Surgery, University of Zaragoza Medical School, University of Zaragoza, Zaragoza, Spain.

*Corresponding authors: victorse@unizar.es, jesus.santamaria@unizar.es

GROUP 1	GROUP 2	GROUP 3	GROUP 4	GROUP 5
Control	100 µg Pt-Exos$^{U251-MG}$	100 µg Pt-Exos$^{U251-MG}$	Cisplatin	Cisplatin
-	IV	IT	IV	IT

Table S1. Experimental groups included in the *in vivo* study.

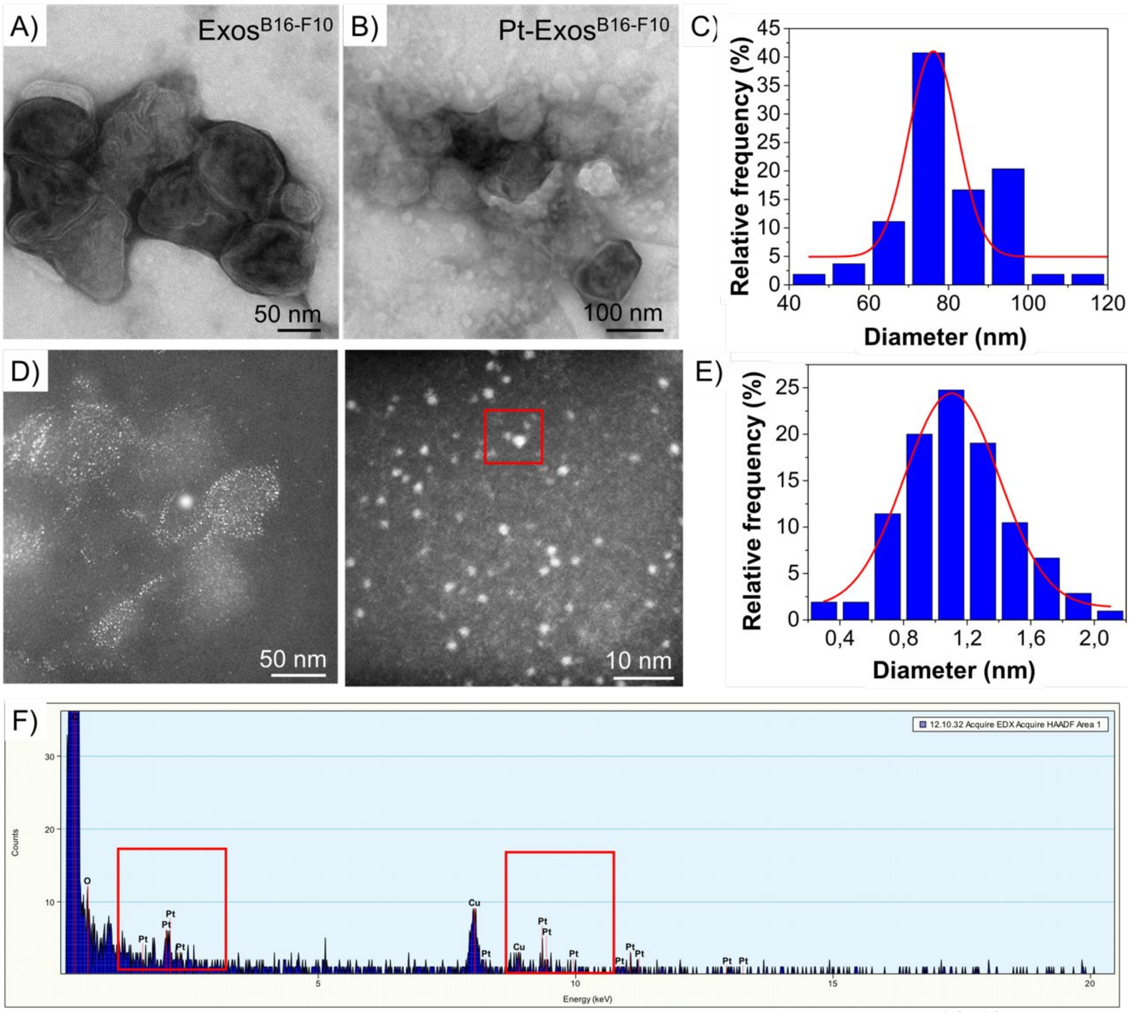

Figure S1. **Characterization of Pt-Exos$^{B16-F10}$**. A) TEM image of Exos$^{B16-F10}$. B) TEM image of Pt-Exos$^{B16-F10}$. C) Size distribution histogram of Pt-Exos$^{B16-F10}$ diameter. D) HAADF-STEM images of Pt-Exos$^{B16-F10}$. E) Size distribution histogram of PtNPs generated within exosomes. F) EDX analysis of the PtNPs synthesized inside the Exos$^{B16-F10}$.

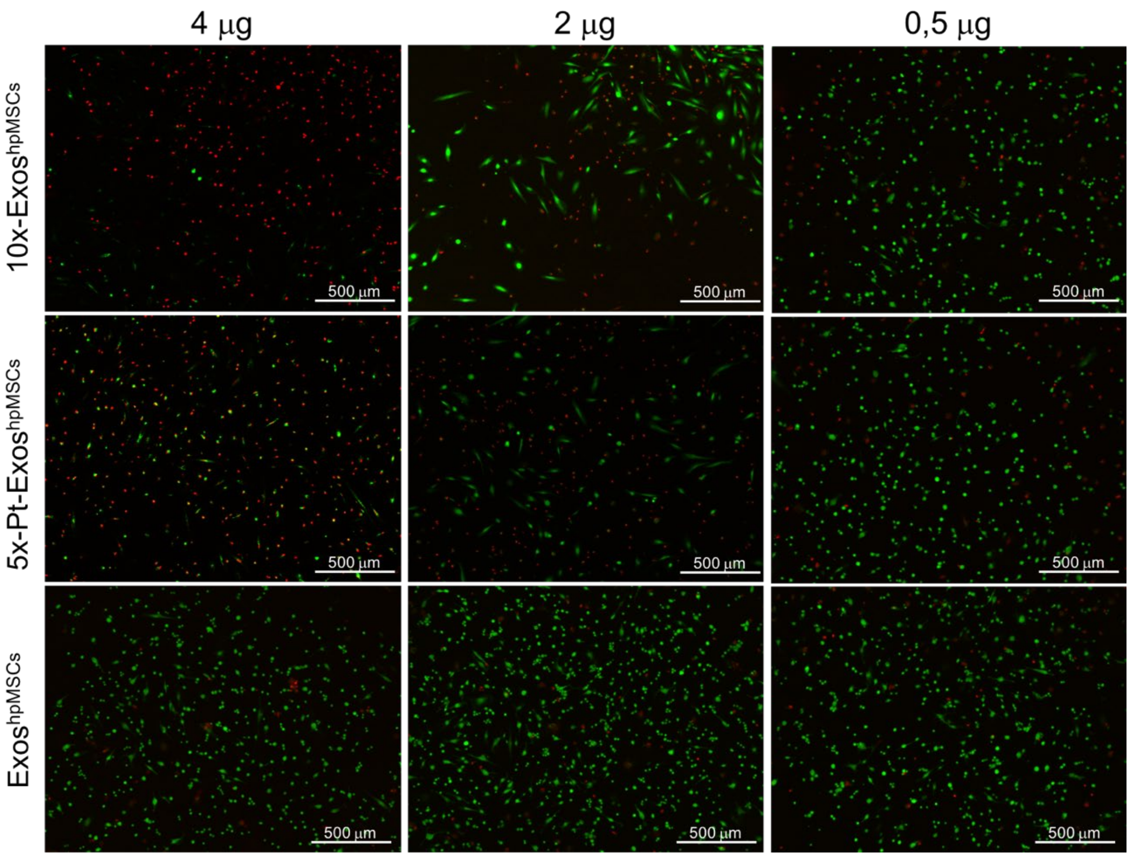

Figure S2. **LIVE/DEAD assay**. Influence of the concentration of Pt-loaded exosomes on cell viability after 48 h of incubation with the corresponding parental cells.

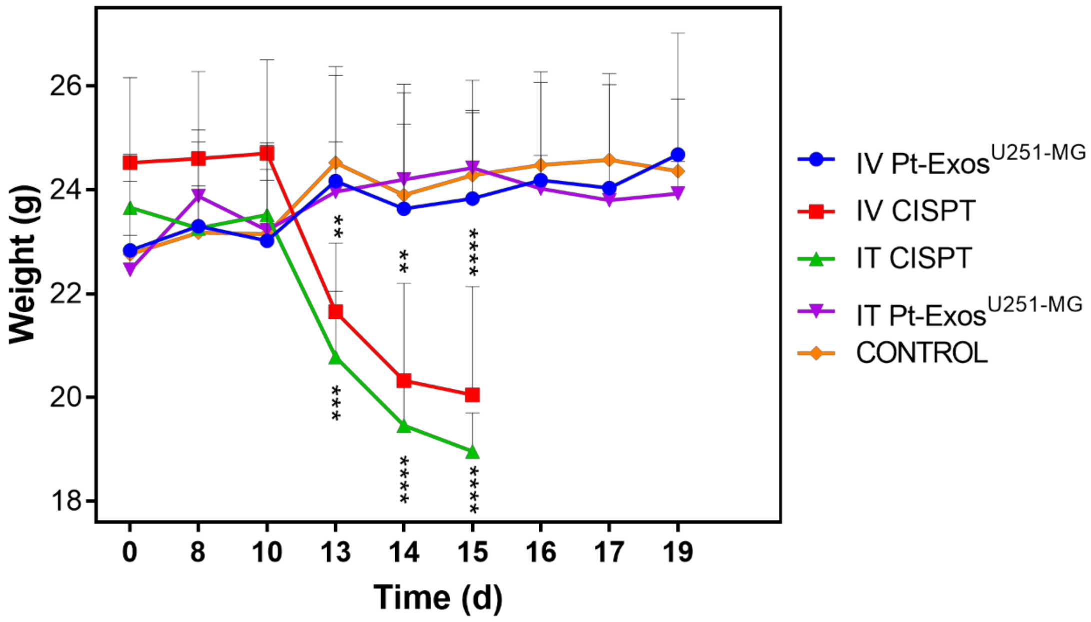

Figure S3. Weight evolution of mice upon cisplatin and Pt-Exos treatment.

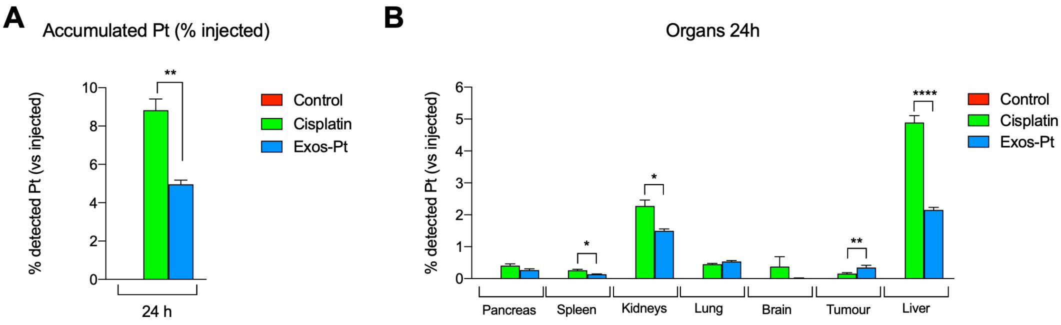

Fig. S4. Biodistribution of Pt 24 h after IV injection of cisplatin and Pt-Exos$_{U251\text{-}MG}$. (A) Amount of detected Pt as a percentage of the total injected per animal (sum of the Pt mass detected in every organ as a percentage of the mass injected) 24 h after treatment. **(B)** Biodistribution of Pt after 24 h in analyzed organs: pancreas, spleen, kidneys, liver, lung, brain and tumor as percentage of the total amount of injected Pt.

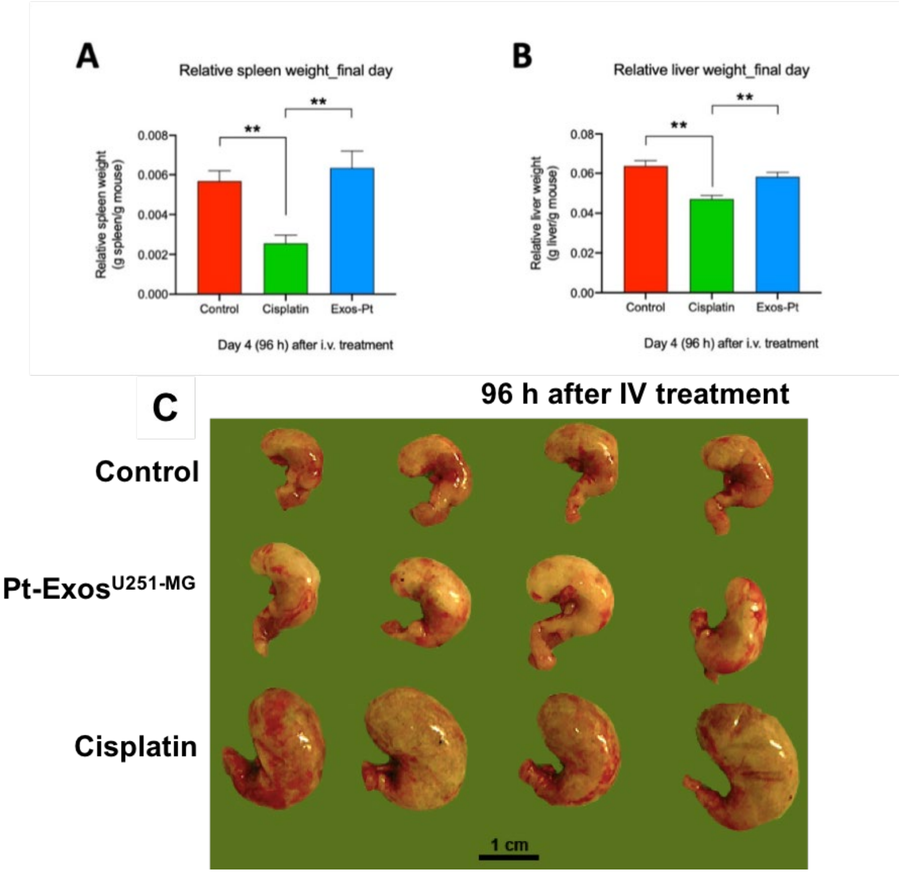

Figure S5. Organic alterations 96 hours after cisplatin intravenous treatment: (A) Reduction of relative spleen weight, (B) Reduction of relative liver weight, (C) enlargement of stomach.

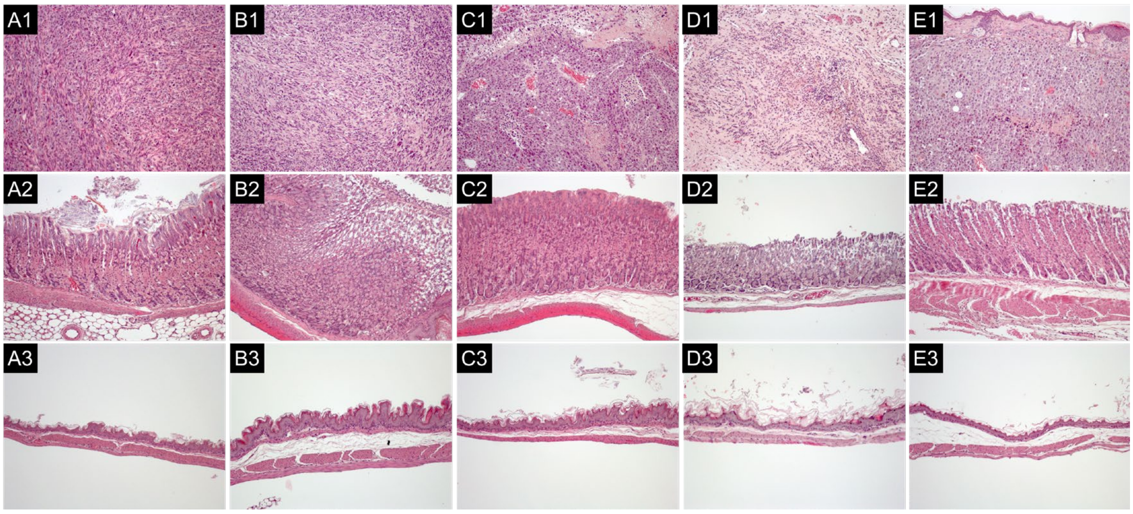

Figure S6. H-E analysis. Tumor (A1), glandular stomach (A2) and non-glandular stomach (A3) from control group. Tumor (B1), glandular stomach (B2) and non-glandular stomach (B3) from IV Pt-Exos treated group. Tumor (C1), glandular stomach (C2) and non-glandular stomach (C3) from IT Pt-Exos treated group. Tumor (D1), glandular stomach (D2) and non-glandular stomach (D3) from IV cisplatin treated group. Tumor (E1), glandular stomach (E2) and non-glandular stomach (E3) from IT cisplatin treated group.
